# Association between household income levels and nutritional intake of allergic children under 6 years of age in Korea: 2019 Korea National Health and Nutrition Examination Survey and application of machine learning

**DOI:** 10.3389/fpubh.2023.1287085

**Published:** 2024-01-08

**Authors:** Seungpil Jeong, Yean Jung Choi

**Affiliations:** ^1^Department of Medical Informatics, College of Medicine, Catholic University of Korea, Seoul, Republic of Korea; ^2^Department of Food and Nutrition, Sahmyook University, Seoul, Republic of Korea

**Keywords:** children, allergic diseases, nutritional intake, household income, machine learning

## Abstract

**Introduction:**

This study investigated the prevalence of allergic diseases in Korean children aged 6 and below, focusing on the interplay between nutritional status, household income levels, and allergic disease occurrence.

**Methods:**

This study used data from the 2019 Korea National Health and Nutrition Examination Survey, a nationwide comprehensive survey, and included a representative sample of 30,382 children under the age of 6 to investigate in detail the relationship between allergic diseases, nutritional intake, and socioeconomic factors. Logistic regression analysis was performed to identify factors associated with allergic diseases, including gender, BMI, eating habits, dietary supplement intake, and nutrient consumption. To predict childhood asthma, 14 machine learning models were compared using the ‘pycaret’ package in Python.

**Results:**

We discerned that 24.7% were diagnosed with allergic conditions like atopic dermatitis, asthma, and allergic rhinitis. Notably, household income exhibited a significant influence, with the lowest income quartile exhibiting higher prevalence rates of asthma, allergic rhinitis, and multiple allergic diseases. In contrast, the highest income quartile displayed lower rates of allergic rhinitis. Children diagnosed with allergic diseases demonstrated compromised intake of essential nutrients such as energy, dietary fiber, vitamin B1, sodium, potassium, and iron. Particularly noteworthy were the deficits in dietary fiber, vitamin A, niacin, and potassium intake among children aged 3–5 with allergies. Logistic regression analysis further elucidated that within low-income families, female children with higher BMIs, frequent dining out, dietary supplement usage, and altered consumption of vitamin B1 and iron faced an elevated risk of allergic disease diagnosis. Additionally, machine learning analysis pinpointed influential predictors for childhood asthma, encompassing BMI, household income, subjective health perception, height, and dietary habits.

**Discussion:**

Our findings underscore the pronounced impact of income levels on the intricate nexus between allergic diseases and nutritional status. Furthermore, our machine learning insights illuminate the multifaceted determinants of childhood asthma, where physiological traits, socioeconomic circumstances, environmental factors, and dietary choices intertwine to shape disease prevalence. This study emphasizes the urgency of tailored nutritional interventions, particularly in socioeconomically disadvantaged populations, while also underscoring the necessity for comprehensive longitudinal investigations to unravel the intricate relationship between allergic diseases, nutritional factors, and socioeconomic strata.

## 1 Introduction

In recent times, the global increase in allergic diseases can be attributed to several factors, including global warming and air pollution, which have contributed to earlier onset of these conditions (1). A recent study has shown a significant decline in the 10-year trend of atopic dermatitis among infants and preschool children, while the trend among school-age children and older adults has exhibited a substantial increase from 2008 to 2017 (2). Specifically, the estimated prevalence of allergic diseases from 2016 to 2017 is as follows: 0.9% (asthma, infants), 2.3% (asthma, preschool children), 4.1% (asthma, school-age children), 2.3% (asthma, adults), 4.1% (asthma, older adult), 9.0% (allergic rhinitis, infants and children), 20.2% (allergic rhinitis, preschool children), 27.6% (allergic rhinitis, school-age children), 17.1% (allergic rhinitis, adults), 6.9% (allergic rhinitis, older adult), 5.9% (atopic dermatitis, infants and children), 11.3% (atopic dermatitis, preschool children), 14.6% (atopic dermatitis, school-age children), 3.9% (atopic dermatitis, adults), 1.6% (atopic dermatitis, older adult) (2). This progression typically initiates with atopic dermatitis during infancy, followed by asthma and allergic rhinitis, underscoring the significance of early interventions for the effective management of allergic diseases in children under 6.

While genetic factors play a pivotal role in shaping allergic conditions, other elements such as the child’s gender, age, body mass index, breastfeeding habits, and nutrient intake also exert significant influences (3). Previous studies have indicated that due to their immature intestinal mucosa and immune function, infants are susceptible to allergic diseases or their exacerbation due to factors like antibiotic consumption and vitamin D deficiencies (4, 5). Intriguingly, the rise in riboflavin and niacin consumption has been correlated with an increase in allergic rhinitis cases among children (6). Thus, a judicious regulation of food intake to mitigate allergenic triggers while enhancing factors that alleviate allergic symptoms becomes paramount.

Given the developmental stage of children under 6, their well-being is substantially shaped by parental practices and dietary choices. Neglectful care and inadequate dietary decisions for children diagnosed with allergies can lead to aggravated or recurrent conditions, potentially giving rise to severe, life-threatening symptoms (1). Conversely, excessively restrictive diets, though intended to manage allergies, might result in nutritional imbalances and hindered growth (7). This underscores the importance of parents holistically managing their children’s diets to align with their growth and developmental requirements.

The escalating trend of westernized diets and lifestyles contributing to a surge in allergic diseases among children has spurred various interventions. Strategies such as promoting breastfeeding and enhancing immune regulation through probiotics to counter the “allergic march” have been advocated (4, 8). However, most research on allergic diseases in children under 6 tends to predominantly focus on living environments, often overlooking the intricate aspects of nutritional intake.

Recognizing this gap, this study aims to illuminate the relationship between the prevalence of allergic diseases and nutritional intake factors in children. Importantly, with household income serving as a determinant in both allergic disease prevalence and nutritional intake, a deeper exploration at this juncture is essential (9–11). Leveraging data from the 2019 Korea National Health and Nutrition Examination Survey, this research endeavors to scrutinize these relationships among children under 6, a pivotal phase for growth and the establishment of dietary patterns. Notably, a comparative analysis of 14 machine learning models was conducted to elucidate the most influential predictor variables for allergic diseases. Ultimately, our objective is to provide empirical insights to shape future nutritional management guidelines tailored to children with allergic diseases, with a particular focus on the role of socioeconomic status.

## 2 Materials and methods

### 2.1 Study participants

This study utilized data from the Korea National Health and Nutrition Examination Survey, which was provided by the Korea Centers for Disease Control and Prevention. The survey divided the country into 192 regions, and probabilistic sampling was conducted from 25 households within each region. Data collection occurred annually. Health statistics were computed by analyzing variables relevant to each subject’s life cycle. Trained researchers visited each household to conduct the survey. For this study, data from the first year of the 8th survey period (2019) were employed.

### 2.2 Participant selection

The study included a total of 30,382 children, out of the initial pool of 32,985 children under the age of 6. Children who had not provided any response regarding allergic disease diagnosis, as well as those with missing values in nutrient intake data, were excluded from the analysis.

### 2.3 Variables

The comprehensive items within the Korea National Health and Nutrition Examination Survey encompass health questionnaires, checkup surveys, and nutrition intake surveys. In this investigation, we focused on health questionnaire and nutrition intake survey data pertaining to children aged below 6 years. Demographic attributes for children encompassed age, gender, residential area, and household income. The prevalence of allergic diseases was ascertained through the diagnosis of atopic dermatitis, asthma, or allergic rhinitis, with categorization into ‘yes’ for diagnosed cases and ‘no’ for non-diagnosed cases. Regarding physical characteristics, the child’s height, weight, birth weight, and Body Mass Index (BMI) were employed for analytical purposes.

In this study, we utilized data from the Korea National Health and Nutrition Examination Survey, which did not provide extensive information specifically on food allergies. Consequently, we redirected our research focus toward exploring the broader association between various allergic diseases and key factors such as nutritional status and household income levels, instead of limiting our investigation to food allergies alone. To accurately assess the energy and nutrient intake relevant to our study’s scope, we employed the 24-h recall method, a standard dietary assessment tool. The energy contribution of macronutrients was computed by multiplying carbohydrate intake by 4 kcal, protein intake by 4 kcal, and fat intake by 9 kcal, subsequently dividing by daily energy intake. Macro-minerals including calcium, phosphorus, sodium, and potassium, alongside micro-mineral intake, primarily iron, were subjected to analysis. In the realm of vitamins, the investigation considered vitamin A, vitamin B1, vitamin B2, niacin, and vitamin C. Additionally, the study incorporated saturated fatty acids, cholesterol, sugar, and dietary fiber for further analysis.

Within the scope of nutritional intake factors, the analysis encompassed breastfeeding, formula feeding, and the introduction of weaning foods. Furthermore, data on breakfast frequency, dining out frequency, dietary therapeutic measures, and dietary supplement usage were examined. Notably, data collection pertaining to breastfeeding and formula feeding status, initiation of weaning foods, and birth weight were limited to children under 3 years of age, with subsequent analysis restricted to this age cohort.

### 2.4 Data analysis

The collected data underwent analysis using SAS 9.4 (SAS Institute Inc., Cary, NC, USA). The characteristics of children aged under 6 years and the incidence frequency of allergic diseases were discerned. An in-depth analysis was performed to explore the associations between demographic attributes, health behaviors, nutrient intake, and the occurrence of allergic diseases among the subjects. To uncover the factors influencing allergic diseases in children, a multiple-sample logistic regression analysis was conducted.

Furthermore, a comprehensive set of 14 machine learning tools was employed to classify instances of allergic diseases. These tools encompassed K Neighbors, Light Gradient Boosting, Extra Trees, Decision Tree, Random Forest, Gradient Boosting, Quadratic Discriminant Analysis, Logistic Regression, Ada Boost Classifier, Linear Discriminant Analysis, Ridge Classifier, Dummy Classifier, SVM-Linear Kernel, and Naive Bayes. The input variables used for classification comprised height, weight, BMI, age, sex, residence, household income (quartile), subjective health awareness, breakfast frequency, dining out frequency, meal therapy, and dietary supplement usage.

The Python package employed for this analysis was ‘pycaret,’ a versatile tool for the comparison of diverse machine learning models.

### 2.5 Cluster-based predictive modeling for asthma diagnosis: a comprehensive approach using imputation, normalization, and stratified cross-validation

Our study was designed to predict the likelihood of asthma diagnosis within a substantial cohort of 198,964 individuals stratified across four distinct clusters. The dataset underpinning our analysis was comprehensive, comprising 16 attributes tailored to represent the unique characteristics of each cluster. In preparing the data for analysis, meticulous preprocessing steps were undertaken. We addressed the issue of missing values by employing simple imputation techniques, where missing numerical data were replaced with mean values and missing categorical data with the mode. Furthermore, we utilized the Yeo-Johnson transformation method to normalize the data, applying a z-score approach to scaling. To maintain the rigor of our analysis and avoid multicollinearity, we reduced the feature set from 16 to 15 by removing those with a correlation coefficient above 0.95.

The subsequent phase of our methodology involved the training and evaluation of predictive models. A 10-fold StratifiedKFold cross-validation strategy was implemented to evaluate the models, ensuring a robust training protocol and reliable performance assessment. Each model was carefully configured with a set of hyperparameters specifically chosen to be congruent with the underlying algorithm, followed by an optimization process to fine-tune these parameters to their optimal values. In the final verification stage, a portion of the data, ranging from 20 to 25%, is used for testing. This approach enabled us to validate the predictive accuracy of our models on an independent dataset, thereby confirming their generalizability and effectiveness in predicting asthma diagnoses within the given population (12, 13).

## 3 Results

### 3.1 Comparison of general characteristics

Table 1 illustrates the variations in general characteristics based on the presence or absence of diagnosed allergic diseases. The study encompassed 30,382 subjects, with an allergic disease prevalence of 24.7%. Allergic rhinitis emerged as the most prevalent condition, accounting for 76.4%, followed by atopic dermatitis at 27.3%, diagnoses of two or more concurrent allergic diseases at 11.1%, and asthma at 7.5%. Significant distinctions were evident between the two groups concerning factors such as age, gender, height, BMI, residential area, and household income in relation to allergic disease diagnoses.

**Table 1:** General characteristics of the subjects.

	Allergic diseases		value of *p*
Variables	Yes (*n* = 7,502)	No (*n* = 22,880)	
Age (yrs.)	4.2 ± 1.4	3.8 ± 1.6	<0.0001^1^
Age (yrs.)			<0.0001^2^
1–2	226 (3.0%)	1924 (8.4%)	
2–3	812 (10.8%)	3,535 (15.5%)	
3–4	1,296 (17.3%)	4,718 (20.6%)	
4–5	1,491 (19.9%)	2,470 (18.7%)	
≥5	3,677 (49.0%)	8,433 (36.9%)	
Sex (n (%))			<0.0001
Male	4,477 (59.7%)	11,575 (50.6%)	
Female	3,025 (40.3%)	11,306 (49.4%)	
Height (cm)	107.7 ± 11.6	104.8 ± 13.1	0.0019
Weight (kg)	19.0 ± 4.4	18.0 ± 4.9	0.4186
BMI (kg/m^2^)	16.1 ± 1.3	16.1 ± 1.6	0.0113
Region (n (%))			0.1982
Urban	5,091 (67.9%)	15,343 (67.1%)	
Rural	2,411 (32.1%)	7,537 (32.9%)	
Family income level (n (%))			<0.0001
Low	272 (3.6%)	1,059 (4.6%)	
Middle low	2,697 (36.0%)	7,923 (34.6%)	
Middle high	3,063 (40.8%)	7,693 (33.6%)	
High	1,470 (19.6%)	6,205 (27.1%)	
Allergic diseases (n (%))			
Atopic dermatitis	2,048 (27.3%)		<0.0001
Asthma	560 (7.5%)		<0.0001
Allergic rhinitis	5,730 (76.4%)		<0.0001
Combined^3^	836 (11.1%)		<0.0001

^1^Different between two groups at *α* = 0.05 by ANCOVA test. ^2^Different between two groups at *α* = 0.05 by chi-square test. ^3^Combined; have 2 or more allergic diseases.

### 3.2 Characteristics related to dietary habits

Table 2 delineates attributes linked to dietary behaviors contingent on allergic disease diagnoses. Substantial divergences were observed between the two groups in terms of breakfast frequency, dining out frequency, dietary therapeutic measures, and dietary supplement consumption. Additionally, noteworthy discrepancies were noted regarding breastfeeding, formula feeding, birth weight, and initiation time of weaning foods within the subset of children aged 1–3 years who received allergic disease diagnoses.

**Table 2:** Dietary habits variables of the subjects.

	Allergic diseases		value of *p*^1^
Variables	Yes (*n* = 7,502)	No (*n* = 22,880)	
Eating breakfast (n (%))			<0.0001
5–7 times/w	6,582 (87.7%)	19,698 (86.1%)	
3–4 times/w	365 (4.9%)	1,638 (7.2%)	
1–2 times/w	471 (6.3%)	813 (3.6%)	
None	84 (1.1%)	731 (3.2%)	
Eating out (n (%))			<0.0001
≥1 times/d	2,626 (35.0%)	7,718 (33.7%)	
1–6 times/w	4,809 (64.1%)	14,516 (63.4%)	
<1 time/w	67 (0.9%)	646 (2.8%)	
Diet therapy			<0.0001
Yes	609 (8.1%)	1,486 (6.5%)	
No	6,893 (91.9%)	21,394 (93.5%)	
Eating dietary supplements in a year (n (%))			<0.0001
Yes	6,139 (81.8%)	16,431 (71.8%)	
No	1,363 (18.2%)	6,449 (28.2%)	
**Variables**	**Yes (*n* = 2,334)**	**No (*n* = 10,177)**	**value of *p***^**1**^
Breastfeeding (n (%))			<0.0001
Yes	2,074 (88.9%)	9,600 (94.3%)	
No	260 (11.1%)	577 (5.7%)	
Formula feeding (n (%))			<0.0001
Yes	2,117 (90.7%)	8,582 (84.3%)	
No	217 (9.3%)	1,595 (15.7%)	
Birth weight	3.3 ± 0.5	3.2 ± 0.4	
Time to start eating baby food (month)	5.4 ± 1.2	6.1 ± 2.4	<0.0001

^1^Different between two groups at *α* = 0.05 by chi-square test.

### 3.3 Comparison of allergic disease diagnoses based on household income (quartile)

Table 3 offers a comparative analysis of individuals diagnosed with allergic diseases categorized by household income quartiles. Among those situated in the 1st quartile (lowest income bracket), the highest prevalence rates were identified for asthma, allergic rhinitis, and complex allergic diseases, at 6.5, 20.4, and 6.5%, respectively. In contrast, for subjects belonging to the 4th quartile (highest income bracket), the lowest occurrence was observed for allergic rhinitis, at 16.0%.

**Table 3:** Diagnosis of allergic diseases of the subjects according to household income.

		Household income				
Variables	Total (*n* = 30,382)	Lowest (*n* = 1,331)	Lower middle (*n* = 10,620)	Upper middle (*n* = 10,756)	Highest (*n* = 7,675)	value of *p*^1^
Diagnosis of allergic diseases (n (%))						
Atopic dermatitis	2,048 (6.7%)	0 (0.0%)	630 (5.9%)	894 (8.3%)	524 (6.8%)	<0.0001
Asthma	560 (1.8%)	87 (6.5%)	225 (2.1%)	248 (2.3%)	0 (0.0%)	<0.0001
Allergic rhinitis	5,730 (18.9%)	272 (20.4%)	2,087 (19.7%)	2,145 (19.9%)	1,226 (16.0%)	<0.0001
Combined^2^	836 (2.8%)	87 (6.5%)	245 (2.3%)	224 (2.1%)	280 (3.7%)	<0.0001

^1^Different between groups at *α* = 0.05 by chi-square test. ^2^Combined; have 2 or more allergic diseases.

### 3.4 Comparison of nutritional status

Table 4 presents the contrast in nutritional intake corresponding to allergic disease diagnoses. Concerning energy intake, the allergic disease group exhibited a lower value compared to the normal group, registering at 1,357.98 ± 4.58 kcal. Notably, in terms of carbohydrates and the percentage of carbohydrate energy ratio, dietary fiber, vitamin A, vitamin B1, sodium, potassium, and iron, the allergic disease group displayed lower levels of intake in contrast to the normal group, with statistically significant disparities. Conversely, in the domains of protein, fat, cholesterol, sugar, and calcium, the allergic disease group exhibited higher intake levels relative to the normal group, with these differences also attaining statistical significance. The intake of saturated fatty acids, vitamin B2, niacin, vitamin C, and phosphorus displayed no variance based on allergic disease diagnoses.

**Table 4:** Daily nutrient intakes of the subjects.

	Allergic diseases						value of *p*^1^
Variables	Yes (*n* = 7,502)			No (*n* = 22,880)			
Energy (Kcal)^2^	1,357.98	±	4.58	1,417.93	±	2.61	<0.0001
Carbohydrate (g)	209.69	±	0.35	212.90	±	0.20	<0.0001
Protein (g)	49.25	±	0.11	48.47	±	0.06	<0.0001
Fat (g)	40.15	±	0.13	38.99	±	0.07	<0.0001
Saturated fat (g)	14.72	±	0.06	14.66	±	0.04	0.4137
Cholesterol (mg)	216.76	±	1.40	191.31	±	0.80	<0.0001
Fiber (g)	14.09	±	0.06	14.87	±	0.04	<0.0001
Sugar (g)	57.95	±	0.27	56.58	±	0.15	<0.0001
Vitamin A (μg RAE)	306.81	±	1.95	317.82	±	1.11	<0.0001
Vitamin B1 (mg)	0.80	±	0.00	0.82	±	0.00	<0.0001
Vitamin B2 (mg)	1.18	±	0.00	1.17	±	0.00	0.0637
Niacin (mg)	7.99	±	0.03	8.00	±	0.02	0.7870
Vitamin C (mg)	63.51	±	0.71	63.06	±	0.40	0.5769
Calcium (mg)	452.29	±	2.04	446.78	±	1.16	0.0194
Phosphorus (mg)	787.01	±	1.77	787.82	±	1.01	0.6914
Sodium (mg)	1,748.27	±	7.06	1,767.71	±	4.02	0.0172
Potassium (mg)	1,681.41	±	4.83	1,726.90	±	2.75	<0.0001
Iron (mg)	5.81	±	0.03	5.89	±	0.02	0.0162
Energy distribution							
% Carbohydrate	60.40	±	0.09	61.40	±	0.05	<0.0001
% Protein	14.19	±	0.03	13.92	±	0.02	<0.0001
% Fat	25.41	±	0.08	24.68	±	0.04	<0.0001

^1^Different between two groups at *α* = 0.05 by ANCOVA test adjusted for age, gender, energy (except energy). ^2^Age and sex-adjusted least squares means (LSmeans).

### 3.5 Assessment of dietary quality

In evaluating diet quality, the results revealed that the allergic disease group registered lower Nutrient Adequacy Ratio (NAR) values for dietary fiber, vitamin A, and calcium in comparison to the normal group. Additionally, these values were below 1. It’s worth noting that although not statistically significant, the Mean Adequacy Ratio (MAR) value was also notably low (Table 5). Furthermore, the Index of Nutritional Quality (INQ) demonstrated that the allergic disease group exhibited diminished intakes of dietary fiber, vitamin A, niacin, and potassium in comparison to the normal group, with values falling below 1 (Table 6).

**Table 5:** NAR^1^ and MAR^2^ of the subjects aged 3–5 years.

	Allergic diseases						value of *p*^3^
Variables	Yes (*n* = 4,676)			No (*n* = 12,039)			
Protein^4^	1.93	±	0.01	1.92	±	0.01	0.1435
Fiber	0.69	±	0.01	0.73	±	0.00	<0.0001
Vitamin A	0.96	±	0.01	0.99	±	0.01	0.0025
Vitamin B1	1.58	±	0.01	1.63	±	0.01	0.0002
Vitamin B2	1.93	±	0.01	1.82	±	0.01	<0.0001
Niacin	1.13	±	0.01	1.13	±	0.00	0.9589
Vitamin C	1.44	±	0.02	1.41	±	0.01	0.2552
Calcium	0.70	±	0.00	0.68	±	0.00	<0.0001
Phosphorus	1.38	±	0.01	1.38	±	0.00	0.5554
Sodium	1.71	±	0.01	1.77	±	0.01	<0.0001
Potassium	0.68	±	0.00	0.69	±	0.00	0.1907
Iron	0.82	±	0.01	0.82	±	0.00	0.4318
MAR	1.24	±	0.01	1.25	±	0.00	0.9664

^1^NAR, Nutrient adequacy ratio. ^2^MAR, Mean adequacy ratio. ^3^Different between two groups at *α* = 0.05 by ANCOVA test adjusted for age, gender. ^4^Age and sex-adjusted least squares means (LSmeans).

**Table 6:** INQ^1^ of the subjects aged 3–5 years.

	Allergic diseases						value of *p*^2^
Variables	Yes (*n* = 4,676)			No (*n* = 12,039)			
Protein^3^	1.98	±	0.01	1.95	±	0.00	0.0002
Fiber	0.70	±	0.00	0.74	±	0.00	<0.0001
Vitamin A	0.96	±	0.01	1.02	±	0.00	<0.0001
Vitamin B1	1.62	±	0.01	1.68	±	0.01	<0.0001
Vitamin B2	1.95	±	0.01	1.88	±	0.01	<0.0001
Niacin	1.14	±	0.01	1.15	±	0.00	0.0415
Vitamin C	1.42	±	0.02	1.44	±	0.01	0.5622
Calcium	0.71	±	0.00	0.70	±	0.00	0.0004
Phosphorus	1.41	±	0.00	1.41	±	0.00	0.0870
Sodium	1.79	±	0.01	1.80	±	0.01	0.3422
Potassium	0.69	±	0.00	0.70	±	0.00	0.0002
Iron	0.83	±	0.00	0.84	±	0.00	0.0911

^1^INQ, Index nutritional quality. ^2^Different between two groups at *α* = 0.05 by ANCOVA test adjusted for age, gender. ^3^Age and sex-adjusted least squares means (LSmeans).

### 3.6 Odds ratios for factors impacting allergic disease presence or absence

Following logistic regression analysis, which involved entering factors demonstrating significant differences in accordance with allergic disease diagnoses, several trends emerged. Among these, in the context of household income Q1 (Lowest), gender, BMI, dining out frequency, and dietary supplement usage exhibited an elevated risk of allergic disease (Table 7). Specifically, girls exhibited a 3.075-fold higher risk compared to boys (95% CI, 2.333–4.053). With an increase in BMI, the risk rose by 1.247 times (95% CI, 1.147–1.356). A pronounced elevation in risk was noted when dining out frequency exceeded once a day, standing at 13.045 times (95% CI, 9.198–18.500) compared to dining out 1–6 times weekly. The risk associated with dietary supplement intake was 4.430 times higher (95% CI, 3.186–6.160). Conversely, within the context of household income Q4 (Highest), an increase in BMI was linked to a 0.962-fold decrease in risk (95% CI, 0.928–0.997). Similarly, a 1.776-fold reduction in risk (95% CI, 1.543–2.043) was observed with dietary supplement consumption.

**Table 7:** Odds ratio of various factors on allergic diseases in the lowest income level.

	Household income level	
Variables	Q1 (Lowest)	Q4 (Highest)
	OR (95% CI)	
Age (yrs.)	0.864 (0.793–0.941)	1.012 (0.979–1.047)
value of *p*	0.0008	0.4794
Sex		
Male	1	1
Female	3.075 (2.333–4.053)	0.942 (0.840–1.057)
value of *p*	<0.0001	0.3100
BMI (kg/m^2^)	1.247 (1.147–1.356)	0.962 (0.928–0.997)
value of *p*	<0.0001	0.0347
Eating out (n (%))		
≥1 times/d	13.045 (9.198–18.500)	1.790 (1.593–2.012)
1–6 times/w	1	1
value of *p*	0.9257	0.9529
Urban		
Yes	1.210 (0.927–1.580)	1.122 (0.981–1.284)
No	1	1
value of *p*	0.1608	0.0918
Eating dietary supplements in a year		
Yes	4.430 (3.186–6.160)	1.776 (1.543–2.043)
No	1	1
value of *p*	<0.0001	<0.0001
Vitamin B1	0.090 (0.054–0.150)	1.997 (1.683–2.370)
value of *p*	<0.0001	<0.0001
Vitamin B2	0.191 (0.133–0.274)	1.037 (0.937–1.148)
value of *p*	<0.0001	<0.0001
Niacin	0.875 (0.846–0.905)	1.037 (1.020–1.055)
value of *p*	<0.0001	<0.0001
Iron	1.037 (0.994–1.082)	1.150 (1.117–1.183)
value of *p*	0.0952	<0.0001

In our findings, the relationship between vitamin and mineral intake and the risk of allergic diseases varied across different household income quartiles. For Vitamin B1, we observed an increase in the risk of allergic diseases by a factor of 0.090 (95% CI: 0.054–0.150) with increased intake in the lowest income quartile (Q1). In contrast, in the highest income quartile (Q4), this risk escalated by a factor of 1.997 (95% CI: 1.683–2.370) with increased intake. Regarding Vitamin B2, there was an observed increase in the risk of allergic diseases by 0.191 times (95% CI: 0.133–0.274) in the Q1 group correlating with increased intake. In the Q4 group, the risk increased more moderately, by a factor of 1.037 (95% CI: 0.937–1.148). For niacin intake, the risk of developing allergic diseases increased by 0.875 times (95% CI: 0.846–0.905) in the Q1 group and 1.037 times (95% CI: 1.020–1.055) in the Q4 group, demonstrating a trend of higher risk associated with increased intake across income groups. Lastly, in the case of iron, an increase in intake corresponded with an increase in the risk of allergic diseases, from 1.037 times (95% CI: 0.994–1.082) in the Q1 group to 1.150 times (95% CI: 1.117–1.183) in the Q4 group, indicating a similar trend of heightened risk with increased consumption across the income spectrum.

### 3.7 Logistic regression analysis for diet quality evaluation

Table 8 portrays the outcomes of logistic regression analysis investigating the relationship between allergic disease diagnoses and diet quality evaluation among subjects aged 3–5 years. Higher Mean Adequacy Ratio (MAR) values appeared to correlate with an increased risk of being diagnosed with allergic disease. Conversely, although not statistically significant, a higher Index of Nutritional Quality (INQ) value seemingly contributed to a reduced risk of allergic disease diagnosis.

**Table 8:** Odds ratio of diagnosis of allergic diseases and diet quality of the subjects aged 3–5 years.

	Allergic diseases		value of *p*
Variables	Yes (*n* = 4,676)	No (*n* = 12,039)	
	OR (95% CI)		
MAR^1^			
4 (Highest)	1.226 (1.155–1.302)	1	<0.0001
INQ^2^			
4 (Highest)	0.949 (0.894–1.008)	1	0.0873

^1^MAR, Mean adequacy ratio. ^2^INQ, Index nutritional quality.

Additionally, an exploration of the relationship between allergic disease diagnoses and diet quality evaluation was conducted, factoring in household income levels (Table 9). If the MAR value was elevated within the context of household income Q1 (lowest), a heightened risk of allergic disease was observed. Similarly, an increase in the MAR value within household income Q4 (highest) led to an increased risk, albeit to a lesser extent. Analogously, a high INQ value within household income Q1 (lowest) was associated with an augmented risk of allergic disease, while within household income Q4 (highest), the risk rose as the INQ value increased, again to a lesser extent.

**Table 9:** Odds ratio of diagnosis of allergic diseases and diet quality in the lowest income level.

	Household income level	
Variables	Q1 (Lowest)	Q4 (Highest)
	OR (95% CI)	
MAR^1^		
3 (Highest)	3.073 (2.201–4.290)	1.775 (1.538–2.048)
value of *p*	<0.0001	<0.0001
INQ^2^		
3 (Highest)	5.293 (3.745–7.483)	2.356 (2.037–2.724)
value of *p*	<0.0001	<0.0001

^1^MAR, Mean adequacy ratio. ^2^INQ, Index nutritional quality.

### 3.8 Predictive analysis of allergic diseases using machine learning: exploration of feature importance across physical, demographic, and lifestyle variables

**Figure 1:**
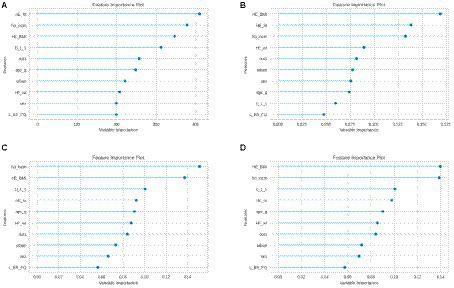
Predicting the occurrence of allergic diseases using Python’s ‘pycaret’ package. Examining the importance of individual features to identify the most influential predictors among children diagnosed with allergic disease and those without. The key predictors of the four models, **(A–D)** included BMI, household income level, and subjective health awareness, indicating the multifaceted nature of these conditions and the potential for advanced predictive tools in healthcare. HE_ht, height; ho_incm, household income; HE_BMI, body mass index (BMI); D_1_1, subjective health awareness; out1, dining out frequency; age_g, age; urban, residential area; HE_wt, weight; L_BR_FQ, breakfast frequency.

In this study, machine learning techniques were harnessed to predict the occurrence of allergic diseases, utilizing an expansive array of physical attributes, demographic data, and health behaviors. By conducting a comparative assessment involving 14 distinct machine learning models and employing Python’s ‘pycaret’ package, the research delved into the significance of individual features to uncover the most influential predictors. The outcome of the comparison among the 14 models is depicted in Figure 1. Among the five models that consistently exhibited performance exceeding 94% across all aspects, an additional step of model optimization was undertaken through hyperparameter tuning. Subsequently, the calculation of feature importance, indicative of their role in predicting the target variables, was conducted. Notably, the K Neighbors Classifier was excluded from this analysis due to its methodology not providing feature importance metrics.

The outcomes gleaned from the four models displayed a coherent pattern. The primary variables contributing significantly across all models were: (A) height, household income, BMI, and subjective health awareness; (B) BMI, height, household income, and weight; (C) household income, BMI, subjective health awareness, and height; (D) BMI, household income, subjective health awareness, and height. These findings underscore the substantial role of BMI, household income (categorized into quartiles), height, and subjective health awareness in determining the likelihood of allergic diseases. Notably, BMI and household income consistently emerged as top predictors, suggesting a strong correlation between these factors and the prevalence of allergic conditions. In contrast, variables related to residential area and dietary habits showed a lesser degree of influence, indicating that while they contribute to the prediction model, their impact is relatively minor compared to the aforementioned key factors.

## 4 Discussion

In our study, we conducted a multifaceted analysis of allergic diseases in children, encompassing demographic, socioeconomic, dietary, and nutritional factors. The significant differences in general characteristics, as shown in Table 1, suggest that age, gender, height, BMI, residential area, and household income are influential in the prevalence of allergic diseases. These findings are critical for pinpointing at-risk populations. Our analysis in Table 2 further underscores the role of dietary habits, including meal frequency and dietary therapeutic measures, in the management of allergic diseases, highlighting the importance of nutritional interventions. Notably, Table 3 reveals a higher prevalence of allergic diseases in lower-income households, pointing to socioeconomic disparities in health outcomes. This is further supported by the nutritional status comparison in Table 4, where children with allergic diseases showed lower intake of essential nutrients but higher intake of proteins, fats, and sugars, suggesting potential areas for dietary modification. Additionally, the assessment of dietary quality in Tables 5, 6, through the NAR, indicates a poorer diet quality in the allergic group, emphasizing the need for improved dietary strategies. Intriguingly, our logistic regression analysis (Table 7) and the evaluation of diet quality (Tables 8, 9) reveal complex interactions between diet, gender, BMI, and allergic disease risk, necessitating further research. Finally, our predictive analysis using machine learning models, as illustrated in Figure 1, highlights the multifaceted nature of allergic diseases, with key predictors such as BMI, household income, and subjective health perception. The results of this study have significant implications for understanding and managing allergic diseases in children. They underscore the importance of considering a wide range of factors, including socioeconomic status, dietary habits, and physical characteristics, in both the prevention and treatment of these conditions. This comprehensive approach underscores the potential of advanced analytical tools in enhancing our understanding and management of allergic diseases in children. This is crucial for developing more effective public health strategies and tailored interventions, especially for populations at high risk due to economic or dietary factors.

While allergic diseases exhibit a high incidence in infants and young children and have a strong correlation with dietary intake, there exists a dearth of systematic research aimed at this age group. In particular, this study centered on unraveling the nexus between nutritional status, household income level, and the prevalence of allergic diseases. A comprehensive survey was conducted encompassing allergic disease prevalence, nutritional intake, and associated risk factors among Korean children under the age of 6. The objective was to facilitate early management and effective interventions for allergic diseases. Our findings indicated a 24.7% prevalence of allergic diseases in children under 6 years, with allergic rhinitis, atopic dermatitis, and asthma ranking in descending order. Notably, recent studies have underscored the sustained high prevalence of allergic rhinitis alongside the stability of asthma’s lower prevalence within the Korean population (2, 14). These results underline the necessity of formulating tailored strategies to prevent allergic diseases across various age brackets in the future. Consistent with existing literature and common perception, our study detected a peak in both asthma and atopic dermatitis during younger ages, typically spanning 5–9 years (15). Furthermore, our findings aligned with a study that witnessed a gradual rise in the prevalence of allergic rhinitis, conjunctivitis, atopic dermatitis, and food allergies among children, albeit with asthma displaying less consistent trends (16).

In our investigation, we identified that the allergic group comprised older boys compared to the normal group. This pattern can be attributed to the “allergy march,” which unfolds as atopic dermatitis, asthma, and allergic rhinitis manifest sequentially with age. These findings are congruent with previous studies that outlined the advanced age of children diagnosed with allergic rhinitis, especially among those developing allergic diseases before the age of 5 (17). In contrast, a prior study focused on children with asthma reported higher incidence rates among boys prior to puberty (18). This diverges from our results, where gender was excluded as a factor in a study concerning allergic disease risk among preschool children (3).

Within our study, the allergic group exhibited greater height and birth weight in comparison to the normal group. This finding resonates with a study exploring nutritional intake in infants afflicted by allergic rhinitis, which also observed heightened height and weight among the allergic rhinitis cohort. This phenomenon seemingly stems from nutritional status or the consumption of nutritional supplements (17). However, considering the similar BMI of our study subjects, it is challenging to definitively attribute this observation to health status or nutritional intake. Given the older age of the allergic group, it is plausible that the growth index was influenced. This correlation mirrors findings from studies examining children with atopic dermatitis and allergic rhinitis, which suggested a lack of connection between BMI and allergic disease (17, 19). Consequently, further research that delves into the intricate relationship between allergic diseases and physical attributes such as height, weight, and BMI is warranted.

Regarding the assessment of eating habits among the study subjects, our findings revealed that the allergic group displayed a higher frequency of eating breakfast 1–2 times a week or skipping breakfast altogether compared to the normal group. The allergic group also exhibited a tendency to eat out frequently and take dietary supplements. These observations align with the results indicating that the allergic disease group had a higher inclination toward snacking and the consumption of instant and fast food, as reported previously (20). Numerous studies have demonstrated that subjects with allergic diseases tend to exhibit poorer eating habits than their normal counterparts. This phenomenon might be attributed not only to food choices but also to societal, cultural, and attitudinal aspects related to eating (18, 21). Consequently, there appears to be a need for educational efforts aimed at enhancing eating habits from early childhood, addressing issues such as picky eating, meal skipping, and mealtime behaviors.

The study also identified a significantly higher frequency of dietary therapy in the allergic disease group as compared to the normal group. This suggests that study participants with allergic diseases likely restricted the consumption of allergenic foods. Noteworthy allergenic foods include eggs, milk, soybeans, buckwheat, meat, fish, shellfish, and peaches (22). A study investigating allergic diseases and antigen sensitization among children and adolescents underscored a high sensitization rate to eggs and milk during infancy (23). Given that this study did not incorporate food allergies into the allergy prevalence category, a comprehensive analysis of dietary causation proves challenging. Therefore, verifying whether dietary choices were determined by medical recommendations or arbitrary decisions becomes essential.

Regarding nutritional intake, our study revealed that the allergic disease group exhibited lower energy intake compared to the normal group. Previous research targeting children aged 4 to 13 established a significant correlation between allergic rhinitis and high-fat, low-carbohydrate diets (24). Conversely, another study examining nutrient intake levels among infants reported higher energy intake among the allergic rhinitis group, yielding conflicting outcomes in comparison to our study (25). However, it’s important to note that the carbohydrate, protein, and lipid intake ratios for both the allergic disease group and the normal group in our study fell within the recommended ranges of 55–65%, 7–20%, and 15–30%, as stipulated in the 2020 Korean Nutritional Intake Standards (26). As children undergo crucial growth and development, maintaining a balanced nutrient intake holds significance. Nonetheless, the more dietary restrictions they encounter due to allergies, the greater the potential impact on nutrient intake, which could contribute to growth impediments (27). Future research will likely be required to delve deeper into the intricate relationship between nutrient intake and the onset of allergic diseases.

Furthermore, this study unveiled that the allergic disease group exhibited lower intake levels of carbohydrates, dietary fiber, vitamin A, vitamin B1, sodium, potassium, and iron compared to the normal group. Particularly noteworthy, the diet quality evaluation revealed that the allergic disease group not only displayed lower INQ values for dietary fiber, vitamin A, and potassium than the normal group but also demonstrated an overall diet quality below 1. This observation gains significance in light of recent efforts to identify biomarkers that aid in categorizing patients for the development and application of targeted treatments for allergic diseases (28–30). To prevent allergic diseases in infants, it becomes imperative to enhance the intestinal environment through dietary fiber consumption (7), activate prebiotics by supplementing Lactobacillus and Bifidus, and ensure adequate vitamin D intake during the first year of life (4, 5). The findings demonstrating the influence on intestinal microbiota composition emphasize the effectiveness of a diet that stimulates intestinal lactic acid bacteria in preventing allergic diseases. This research trend represents a fundamental approach to addressing allergic diseases by activating intestinal lactic acid bacteria rather than merely restricting food antigens, suggesting the need for further exploration and expansion of related studies.

The study’s results also indicate that calcium intake in both groups fell below the recommended range of 500–600 mg. Prior studies have highlighted a correlation between increased prevalence of allergic rhinitis and higher intake levels of protein, calcium, phosphorus, iron, riboflavin, and niacin (17, 20). This trend likely arises from the common practice of restricting milk intake upon allergic disease diagnosis. Calcium holds a pivotal role in keratinocyte differentiation, and insufficient intake can contribute to skin dryness and atopic dermatitis. Therefore, if milk is not consumed, calcium supplementation becomes essential (31). Past research has also indicated lower intake of iron and fat-soluble vitamin retinol in the allergic disease group (32). Differences in nutrient intake from food and supplements might contribute to this variance. Furthermore, although not statistically significant, saturated fatty acid consumption was observed to be higher in the allergic disease group. This contrasts with recommendations to limit saturated fatty acid intake in children with allergic diseases due to potential allergic reactions triggered by fat consumption (19). The study’s results indicate that both groups exceeded the recommended intake of less than 8 g of saturated fat. Given that saturated fat is prevalent in meat and dairy products—known allergens—this observation could reflect dietary management practices. It’s worth noting that prior studies have suggested that saturated fat could weaken the body’s response to allergens, necessitating further exploration of the relationship between allergic diseases and fat intake (7).

This study also revealed significant disparities between the groups in terms of breastfeeding, formula feeding, and the timing of introducing solid foods. Prior research has reported that infants with allergic rhinitis exhibit a higher rate of breast milk intake compared to the normal group, along with an earlier initiation of weaning (17). Breastfeeding serves as a conduit for antigen transmission to infants, and prolonged breastfeeding beyond 6 months is associated with reduced prevalence of atopic dermatitis and asthma among allergic diseases (25). However, conflicting reports exist, suggesting that prolonged breastfeeding might be correlated with an increased risk of atopic dermatitis (8). This is more likely attributed to extended breastfeeding being prevalent in high-risk groups rather than directly causing a rise in allergic disease incidence due to breastfeeding. Other studies have noted breastfeeding’s moderate correlation with reduced asthma risk, without significant influence on atopic dermatitis risk. Moreover, the common practice of introducing solid foods after 6 months stems from parental concerns about food allergies in infants with atopic dermatitis (33). Given the susceptibility of infants’ immature intestinal mucosa and immunity to allergic diseases, early introduction of solid foods before 4 months of age is advised (6, 34). However, research has also suggested that initiating solid foods after 7 months could impact the development of gut microorganisms and immune tolerance, potentially heightening allergic sensitivity and respiratory allergic disease risk. Consequently, a cautious approach is necessary when determining the optimal timing for introducing solid foods (35).

The logistic regression analysis investigating the relationship between allergic diseases and influencing factors yielded notable insights. In low-income families, girls with higher BMI, frequent eating out, dietary supplement intake, decreased intake of vitamin B1, vitamin B2, and niacin were found to have a higher likelihood of experiencing allergic diseases. Moreover, with increasing age, the risk of being in the allergic disease group diminished. Although no significant variance in eating out frequency based on household income was observed, a heightened risk of belonging to the allergic disease group emerged with frequent eating out and consumption of fast food, potentially resulting in nutrient deficiencies due to dietary choices. These findings underscore the significance of balanced nutritional intake and healthy eating habits in preventing the development of allergic diseases among children.

In a prior investigation utilizing data from the 2019 Korea National Health and Nutrition Examination Survey, various factors were identified as risk contributors for allergic rhinitis in infants, including intake of calories, protein, calcium, phosphorus, iron, riboflavin, and niacin (17). Similarly, an examination of the relationship between nutrient intake and atopic dermatitis risk in children under 12 years revealed that excessive fat intake and insufficient or excessive vitamin C intake impacted the likelihood of atopic dermatitis (19). Additionally, an analysis of antigen sensitization patterns over four decades in Korean children and adolescents demonstrated an increasing prevalence of asthma and allergic rhinitis, along with heightened sensitization to companion animals, crustaceans, and pollen antigens (23). The evolution of children’s dietary habits, influenced by reduced outdoor activity time and augmented exposure to food-related media, further emphasizes the need for continual observation of the intricate link between nutritional intake and allergic diseases (1, 6).

Our study notably elucidated the pronounced impact of income level on the interplay between allergic diseases and nutrition, highlighting a pressing necessity for targeted nutritional interventions, particularly within low-income demographics. Earlier research underscored the association between low individual-level socioeconomic status and a higher disease burden, leading to reduced asthma and atopic dermatitis severity and control (36). As new treatment modalities like biologics gain traction, disparities in accessibility and efficacy have garnered attention, amplifying health inequalities tied to these conditions. What was once characterized as a phenomenon prominent in wealthier nations has evolved to encompass intricate interactions between genetic predisposition and a myriad of environmental exposures, leading to asthma and atopic dermatitis being recognized as significant public health concerns across diverse income strata and geographical regions (15, 37). Hence, it becomes crucial to amass precise epidemiological evidence across the global landscape and within various socio-demographic contexts.

The machine learning outcomes for childhood asthma factors revealed a prominent role for BMI, consistent with previous research linking childhood overweight or obesity with an elevated risk of asthma development (38). This underscores the potential of BMI as a decisive element influencing childhood asthma. Early monitoring and management of BMI could prove pivotal in controlling or averting asthma symptoms in young children. The consistent high ranking of household income as a determinant reinforces the significant impact of socioeconomic factors on childhood asthma, aligning with prior research (39). The connection between respiratory health and socioeconomic indicators underscores the potential influence of socioeconomic disparities on asthma prevalence and severity, emphasizing the necessity for targeted health interventions to address these inequalities. Considering subjective health perception factors, disparities in asthma treatment patterns between children and adults have been highlighted (40). This suggests that caregiver or parent perceptions offer crucial insights into pediatric asthma outcomes. Thus, subjective health perceptions emerge as valuable indicators for assessing asthma risk and developing management strategies for younger age groups. Height and weight’s inclusion in the model resonates with previous studies linking fetal and infant growth patterns to atopy or wheezing disorders in later childhood (41). Consequently, monitoring growth patterns could play a pivotal role in predicting or mitigating asthma risk. The link between diet and asthma identified by the model aligns with global findings, which have hinted at a potential correlation between fast food consumption and asthma (42). This underscores the significance of dietary guidance and awareness, as the types and frequency of consumed foods can impact asthma prevalence. The consideration of residential areas in the model echoes earlier research revealing associations between traffic-related air pollution and childhood asthma and allergies (43). This underscores the pivotal role of environmental factors and their influence on asthma development. Although gender ranked lower in the model, prior research has highlighted its subtle yet influential role in childhood and adolescent asthma (44). This emphasizes that gender, although less dominant compared to other factors, still plays a significant part and should not be disregarded in comprehensive asthma research. Taken together, the machine learning model outcomes, when contextualized with existing literature, accentuate the multifaceted determinants of childhood asthma. Physiological attributes, socioeconomic circumstances, environmental elements, and dietary behaviors all interweave to impact asthma prevalence and severity in children under 6. A comprehensive understanding and management of pediatric asthma necessitates considering the interplay of these diverse factors.

This study sheds light on the prevalence of allergic diseases and their correlations with dietary intake and socioeconomic factors, yet it is important to recognize its limitations and areas necessitating further research. Firstly, the study’s reliance on cross-sectional data from the Korea National Health and Nutrition Examination Survey means that data on children under 6 years with allergic diseases is limited. Furthermore, the scope of investigating breastfeeding and baby food-related factors is confined to children up to age 3, which restricts the ability to conclusively establish relationships. Secondly, the absence of specific information on food allergies in the Korea National Health and Nutrition Examination Survey prompted us to expand our research scope. Instead of focusing solely on food allergies, we explored broader associations among various allergic diseases, nutritional status, and household income. Thirdly, this study predominantly concentrated on nutrient intake and diet quality, as opposed to an in-depth analysis of dietary patterns through Food Frequency Questionnaires (FFQ). This focus means that a more detailed understanding of the nutritional profiles of children under 6 and their correlations with other factors, including dietary allergies, was not achievable due to data limitations. For comprehensive insights, future studies should incorporate a wider array of dietary survey components suitable for all age groups. Fourthly, although family history is a crucial element in the development of allergic diseases, this variable was not captured in the survey data used. Future research should delve deeper into the relationship between nutrient intake and allergic diseases, emphasizing the need for extensive birth cohort studies to investigate the intricate interplay of socioeconomic status, dietary composition, and the development of allergic diseases, enhancing the logical development and coherence of the study’s limitations and future research directions.

This study aimed to uncover factors associated with allergic diseases in children under 6 years of age using data from the 2019 Korea National Health and Nutrition Examination Survey. The primary focus was to establish fundamental data for preventing and managing allergic disease symptoms based on household income levels. Among the participants, 24.7% were diagnosed with allergic diseases, indicating an increased prevalence compared to previous years. Distinctions in age, gender, height, weight, frequency of eating out, and dietary habits emerged between the allergic disease group and the normal group. Nutritional intake analysis revealed deficiencies in energy, carbohydrates, dietary fiber, vitamin A, vitamin B1, sodium, potassium, and iron. Regression analysis confirmed that, particularly in low-income households, girls with higher BMI, frequent eating out, dietary supplement use, and lower intake of vitamin B1, vitamin B2, and niacin were more susceptible to allergic disease diagnosis. This study underscored the pronounced impact of income levels on the nexus between allergic disease and nutrition, highlighting the imperative need for targeted nutritional interventions, particularly in economically disadvantaged populations. Consequently, a consistent provision of dietary education to parents becomes essential to ensure children under 6 receive balanced nutrition, avoiding undue restrictions even when allergic disease cases exhibit heightened sensitivity to specific nutrients. While this study utilized data from the extensive Korea National Health and Nutrition Examination Survey, it is crucial to acknowledge the limitations of cross-sectional design. Therefore, we advocate for prospective birth cohort studies to comprehensively explore the intricate relationship between socioeconomic status, nutritional components in dietary intake, and the occurrence of allergic diseases.

## Data availability statement

The original contributions presented in the study are included in the article/supplementary material, further inquiries can be directed to the corresponding author.

## Ethics statement

Written informed consent was obtained from all participants involved in the KNHANES. Additionally, ethical approval was not required as KNHANES provides anonymous, secondary data that is publicly available for scientific use.

## Author contributions

SJ: Conceptualization, Data curation, Formal analysis, Writing – original draft. YC: Supervision, Writing – review & editing.

## Supplementary material

SUPPLEMENTARY FIGURE 1 Correlation with nutritional profile and other factors. To enhance the visual representation of the correlation matrix, we utilize Python’s Seaborn library to generate a heatmap. This approach offers a color-coded depiction of correlations, simplifying the identification of patterns and relationships among variables.
