# Supplementary figures and images for: Association between household income levels and nutritional intake of allergic children under 6 years of age in Korea: 2019 Korea National Health and Nutrition Examination Survey and application of machine learning
Source: Front Public Health. 2024 Jan 8;11:1287085. doi: 10.3389/fpubh.2023.1287085 (PMC10808989; doi:10.3389/fpubh.2023.1287085)

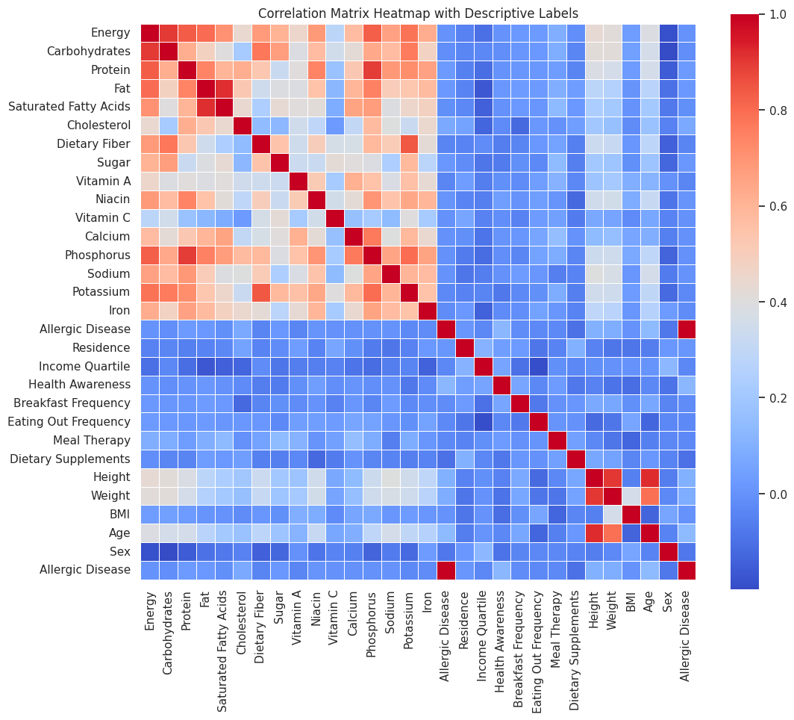

Supplement: SUPPLEMENTARY FIGURE 1 — Correlation with nutritional profile and other factors. To enhance the visual representation of the correlation matrix, we utilize Python’s Seaborn library to generate a heatmap. This approach offers a color-coded depiction of correlations, simplifying the identification of patterns and relationships among variables. [file Image_1.png]
